# Vegetation resistance and resilience to a decade‐long dry period in the temperate grasslands in China

**DOI:** 10.1002/ece3.7866

**Published:** 2021-07-05

**Authors:** Minqi Liang, Ruochen Cao, Kai Di, Daorui Han, Zhongmin Hu

**Affiliations:** ^1^ School of Geography South China Normal University Guangzhou China; ^2^ Southern Marine Science and Engineering Guangdong Laboratory (Zhuhai) Guangdong China

**Keywords:** grassland, long‐term dry period, NDVI, precipitation, resilience, resistance

## Abstract

The duration of climate anomalies has been increasing across the globe, leading to ecosystem function loss. Thus, we need to understand the responses of the ecosystem to long‐term climate anomalies. It remains unclear how ecosystem resistance and resilience respond to long‐term climate anomalies, for example, continuous dry years at a regional scale. Taking the opportunity of a 13‐year dry period in the temperate grasslands in northern China, we quantified the resistance and resilience of the grassland in response to this periodic dry period. We found vegetation resistance to the dry period increased with mean annual precipitation (MAP), while resilience increased at first until at MAP of 250 mm and then decreased slightly. No trade‐off between resistance and resilience was detected when MAP < 250 mm. Our results highlight that xeric ecosystems are most vulnerable to the long‐term dry period. Given expected increases in drought severity and duration in the coming decades, our findings may be helpful to identify vulnerable ecosystems in the world for the purpose of adaptation.

## Graphical abstract

Resistance and resilience of grasslands to a decade‐long dry climate was investigated. Resistance increased with mean annual precipitation (MAP), while resilience increased until at MAP of 250 mm and then decreased slightly. No trade‐off between resistance and resilience was found in xeric grasslands.

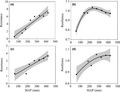

## 1 INTRODUCTION

Grasslands cover more than 30% of the global land surface (Dixon et al., 2014) and provide a series of essential ecosystem services (Häyhä & Franzese, 2014). Stable provision of the services highly depends on the stability of grassland ecosystem functions such as biomass productivity, which are strongly controlled by precipitation (Knapp & Smith, 2001; Harrison et al., 2014). The intensity, frequency, and duration of climate anomalies and extremes are expected to increase throughout the century across the globe (Dai, 2013; Cubasch et al., 2013). Therefore, it is crucial to understand the response of grasslands to climate anomalies. (Ingrisch & Bahn, 2018).

The stability of ecosystems encountering external perturbations has commonly been characterized as resistance and resilience in biomass production (Tilman, 1996). Resistance, defined as the ability of the ecosystem to persist in its normal state during a disturbance (Stuart‐Haëntjens et al., 2018), can be obtained by comparing biomass production in a normal state with that in a disturbed state. Resilience reflects the ability that ecosystem functions return to initial levels after perturbations (Lloret et al., 2011), expressed as the rate of return of a variable (such as biomass production) within a given time after disturbance (Schmid & Pfisterer, 2002). Previous reports on grassland resistance and resistance mainly focused on single‐year extreme drought conditions, with much less attention being paid on ecosystem responses to longer‐term dry periods (e.g., successive several‐year dry climate) (Wilcox et al., 2017; Knapp et al., 2015; Zhao et al., 2015). However, current studies have suggested that drought duration may be as, or perhaps more, influential than drought severity on ecosystem functioning (Evans et al., 2011). It is also predicted that the probability of periodic climatic anomalies that last for several years, such as the drought period from 2000 to 2009 in many regions across the globe (Zhao & Running, 2010; Ponce‐Campos et al., 2013), will increase as a consequence of global climate change (Cubasch et al., 2013). Therefore, it is critical to clarify the resistance and resilience of grassland in response to adverse periodic climate anomalies.

Theory (Grime et al., 2000) and a few case studies (Gazol et al., 2018) reported a trade‐off between resistance and resilience for biomass production (Ruppert et al., 2015), that is, the higher resistance of grasslands may have an inhibitory effect on the recovery of productivity following drought (Craven et al., 2018). However, our knowledge on the relationship between resistance and resilience is still very limited for three reasons. First, the number of relevant studies is small. A meta‐analysis by Matos et al. (2020) suggested that most studies focused on resistance, with very few focusing on resistance and resilience simultaneously. Second, the findings supporting the trade‐off were mostly derived from extreme drought in single years, with seldom from several continuous dry years (Gazol et al., 2018). Third, previous studies were mostly conducted in single ecosystems (e.g., temperate broadleaf forests in the north‐eastern USA; Gazol et al., 2017), and thus, our knowledge on the spatial patterns along climate gradients is still very limited (Matos et al., 2020).

Taking the opportunity of a 13‐year dry period (1999–2011) in the temperate grasslands in northern China, here we quantified the resistance and resilience of the ecosystems in response to this periodic dry period. With the data of the Normalized Difference Vegetation Index (NDVI), a proxy of grassland biomass production, this study aims to address the following research questions: (i) How resistance and resilience in the temperate grasslands vary along a precipitation gradient during the long‐term dry period? (ii) Is there a trade‐off between resistance and resilience in the temperate grasslands along the precipitation gradient?

## 2 METHOD AND MATERIALS

### 2.1 Study area

**FIGURE 1:**
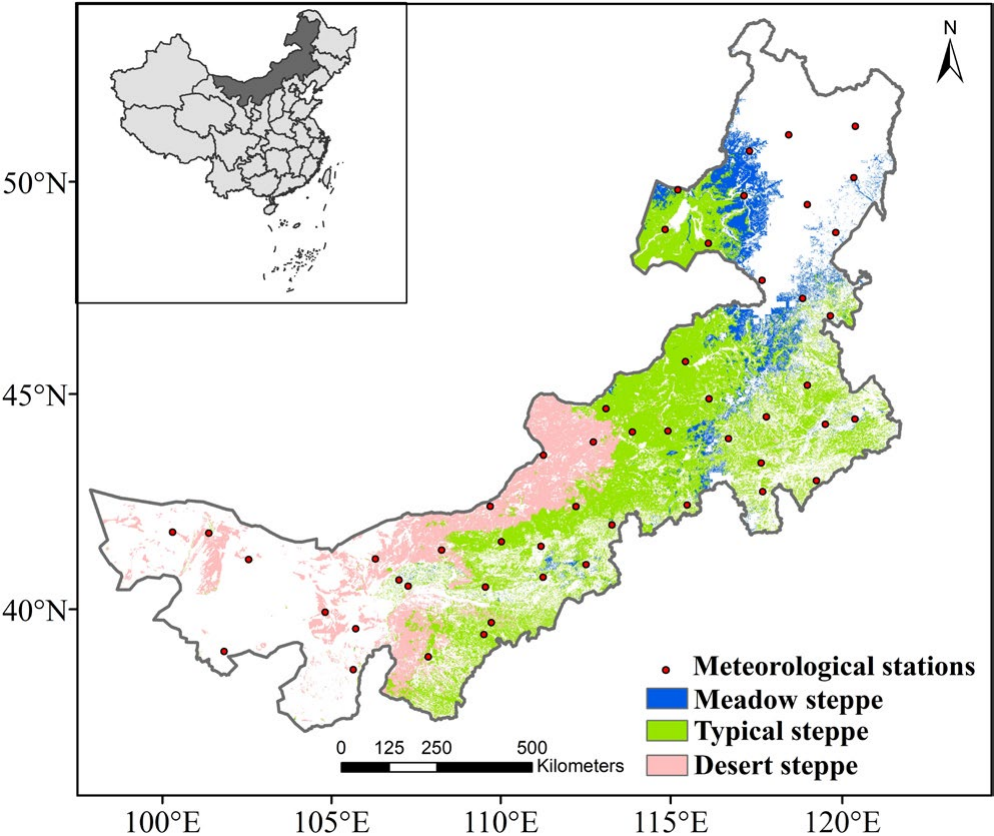
Spatial distribution of grasslands in Inner Mongolia

The temperate steppe of Inner Mongolia is located on the Inner Mongolia Plateau and extends from northeast of China, which covers an area of about 22% of the total area Chinese grassland. This grassland is influenced by the temperate continental climate, distributed along with mean annual precipitation (MAP) gradient from 40 mm to 500 mm, mean annual temperature (MAT) from −3℃ to 9℃. The growing season occurs from April to October. Rain falls in a shorter rainy season in summer, and growing season precipitation accounts for 80% of the annual precipitation in our study area (Guo et al., 2015). Snow only accounts for less than 10% of annual precipitation. From southwest to northeast, precipitation shows an increasing trend, while the temperature is the opposite. Along the precipitation range from dry to wet, grasslands here are divided into three major types, xeric desert steppe, semi‐arid typical steppe, and mesic meadow steppe (Figure 1; Guo et al., 2012). Desert steppe mainly occurs in the driest areas of Inner Mongolia where MAP only ranges from 0 to 200 mm, which is dominated by short xerophytic species (mainly semishrubs and short shrubs) with a height of 10–25 cm and lowest biodiversity (5–10 maximum species in 1 m^2^). Dominant species in typical steppe with MAP is approximately between 200 and 400 mm, is xerophytic‐tufted perennial grasses, and plant communities here are taller (14–35 cm) with diversity (12–15 maximum species in 1 m^2^). Located in the wettest areas of Inner Mongolia, the typical steppe with MAP ranging from approximately 300–600 mm is the most productive grassland of these three types. It is characterized by much higher (35–50 cm) rhizomatous perennial grasses, and plants here with diversity (15–25 maximum species in 1 m^2^) are very common (Hu et al., 2018). The transformation of soil types from brown calcic soil to chestnut and chernozem soil corresponds to the grassland type.

### 2.2 Datasets

The Global Inventory Modeling and Mapping Studies (GIMMS, https://ecocast.arc.nasa.gov/data/pub/gimms/) NDVI datasets with a spatial resolution of 0.083° from 1983 to 2013 were used to quantify the vegetation status. The NDVI was 15‐day composites, and thus, annual NDVI was calculated as the mean of all the 15‐day NDVI of each year.

The station‐specific precipitation data from 51 meteorological stations in Inner Mongolia (locations of these stations were showed as points in Figure 1) from 1982 to 2013 was obtained from the China Meteorological Data Service Center website (http://data.cma.cn/en). A thin plate smoothing spline interpolation method in the Anuspline software package was used to interpolate the station‐specific data with daily site data (Hutchinson, 1995). We summed them to annual values in the calendar year and then resampled to the same spatial resolution as the NDVI data.

### 2.3 Metrics of resistance and resilience

**FIGURE 2:**
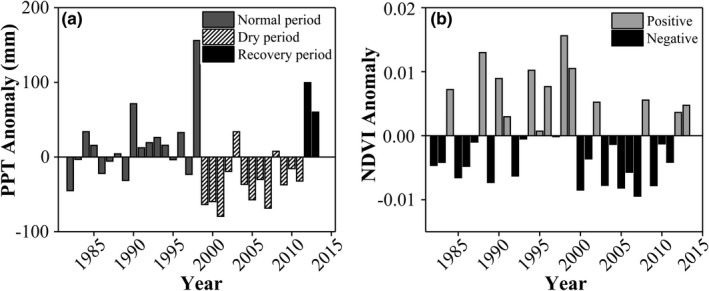
Time series of precipitation anomaly (a) and NDVI anomaly (b) during 1982–2013. The anomaly was calculated as the difference between annual precipitation (or NDVI) and mean annual precipitation (or NDVI) of the whole study period (1982–2013)

The degrees of reduction in precipitation and NDVI in a dry period were calculated for each pixel as:

(1) $$\text{Rain reduction}=\left(P_{\text{normal}}‐P_{\text{dry}}\right)/P_{\text{normal}}$$

(2) $$\text{NDVI reduction}=\left({\text{NDVI}}_{\text{normal}}‐{\text{NDVI}}_{\text{dry}}\right)/{\text{NDVI}}_{\text{normal}}$$

where $P_{\text{normal}}$ and $P_{\text{dry}}$ are the mean precipitation in normal period and dry period, respectively, which are determined from annual precipitation anomaly (Figure 2); ${\text{NDVI}}_{\text{normal}}$ and ${\text{NDVI}}_{\text{dry}}$ are separately the mean NDVI during normal period and dry period.

Isbell et al. (2015) adopted Equation (2) to quantify vegetation resistance. In our study, the degrees of rainfall reduction between sites were different. To exclude the compounding effects of the different effects of rainfall reduction, we quantified resistance by normalizing Equation (2) with the degree of rainfall reduction for each site:

(3) Resistance=Rain reduction/NDVI reduction.

A high resistance suggests that NDVI reduction is relatively smaller than precipitation and vice versa. Resilience was quantified by the ratio of the NDVI during the recovery period to the NDVI during the dry period (Van Ruijven & Berendse, 2010):

(4) $$\text{Resilience}={\text{NDVI}}_{\text{post}}/{\text{NDVI}}_{\text{normal}}$$

where NDVI_post_ is the mean NDVI in the recovery period. Here, we used the mean of the two years (2012–2013) of NDVI after the dry period as NDVI_post_. A higher value of resilience value indicates that vegetation has a higher recovery rate after a dry period. In addition, grassland types (e.g., meadow steppe, typical steppe, and desert steppe) for each pixel in Inner Mongolia were extracted from a Chinese Grassland Classification (http://www.geodata.cn). However, using the grassland map alone cannot guarantee excluding all pixels affected by human activities. Land use has been changed greatly during the recent decades, for example, afforestation with the implements of national ecological restoration projects (Huang et al., 2013; Cai et al., 2020), and reclamation (Li et al., 2019). In addition, the resolution of newer land cover and use products is too coarse to identify the area affected by these human activities. Therefore, to exclude the pixels impacted by human activities, the reduction of NDVI < 1% was excluded for analysis. In addition, pixels without rain reduction were also excluded because here we focus on the impacts of dry condition. To better showing the spatial pattern of resistance and resilience along MAP gradient, we divided all pixels into 8 groups with 50 mm intervals, and then, we averaged each group. We fitted the relations with linear functions at first. If *p* > 0.05 for the fitted linear function, we tried other functions, that is, exponential, quadratic, and cubic and selected the one with the highest *R* ^2^.

## 3 RESULTS

During the whole study period (1982–2013), ecosystems in the study region experienced a 13‐year below‐average dry period, that is, 1999–2011 (11.5% or 35 mm/yr below average; Figure 2a). In comparison, the period preceding this dry period (i.e., 1982–1998) was a normal period (4.9% or 15 mm/year above average), and the period following this dry period (i.e., 2012–2013) with relatively higher precipitation than average was considered as the recovery period. NDVI anomaly showed a similar temporal dynamics to the precipitation anomaly, with obvious reduction during the dry period (Figure 2b). This suggested that this long‐term dry climate condition constrained vegetation growth.

**FIGURE 3:**
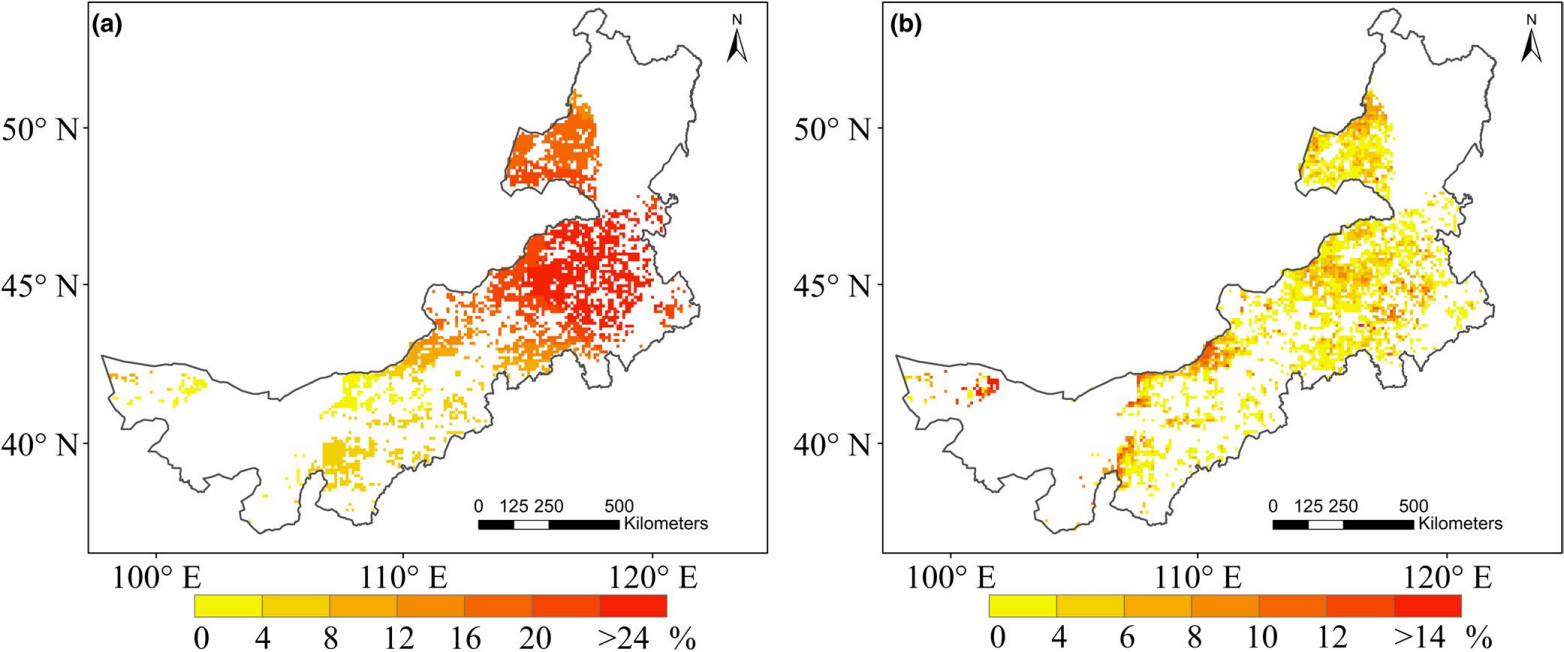
Spatial patterns of the rain reduction (a) and NDVI reduction (b) during the decade‐long dry period

**FIGURE 4:**
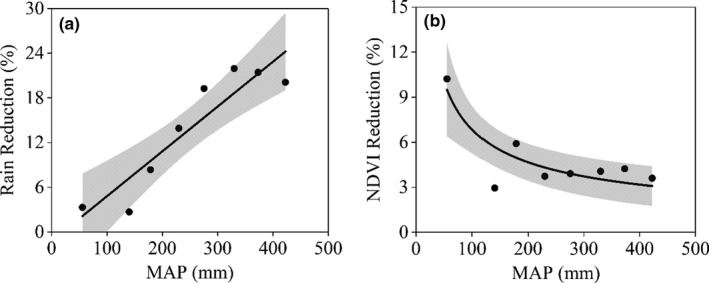
Degree of reduction in rainfall (a) and NDVI (b) along mean annual precipitation gradient (*p* < 0.05). The shaded area depicts the 95% credible interval of the fitting

The reduction of precipitation during the dry period in comparison with the predry period illustrated an obvious spatial pattern in the study area (Figure 3a). For example, the degree of precipitation reduction increased obviously from west to east, that is, the reduction was small in the dry desert ecosystems but large in more humid meadow steppe regions (Figure 1). In comparison with precipitation, except for some xeric areas in the western region, the reduction of NDVI during the dry period was overall low across the study region (Figure 3b). In general, precipitation reduction degree increased with the MAP gradient (Figure 4a; *p* < .05; *R* ^2^ = 0.83). Specifically, the PPT reduction was relatively low when MAP <150 mm and showed a weak decline when MAP >350 mm (Figure 4a). In contrast, NDVI did not illustrate a trend of reduction along the MAP gradient (fluctuated around 4%; *p* < 0.05; *R* ^2^ = 0.67) except for some obvious reductions in the dry end (MAP < 100 mm; Figure 4b).

**FIGURE 5:**
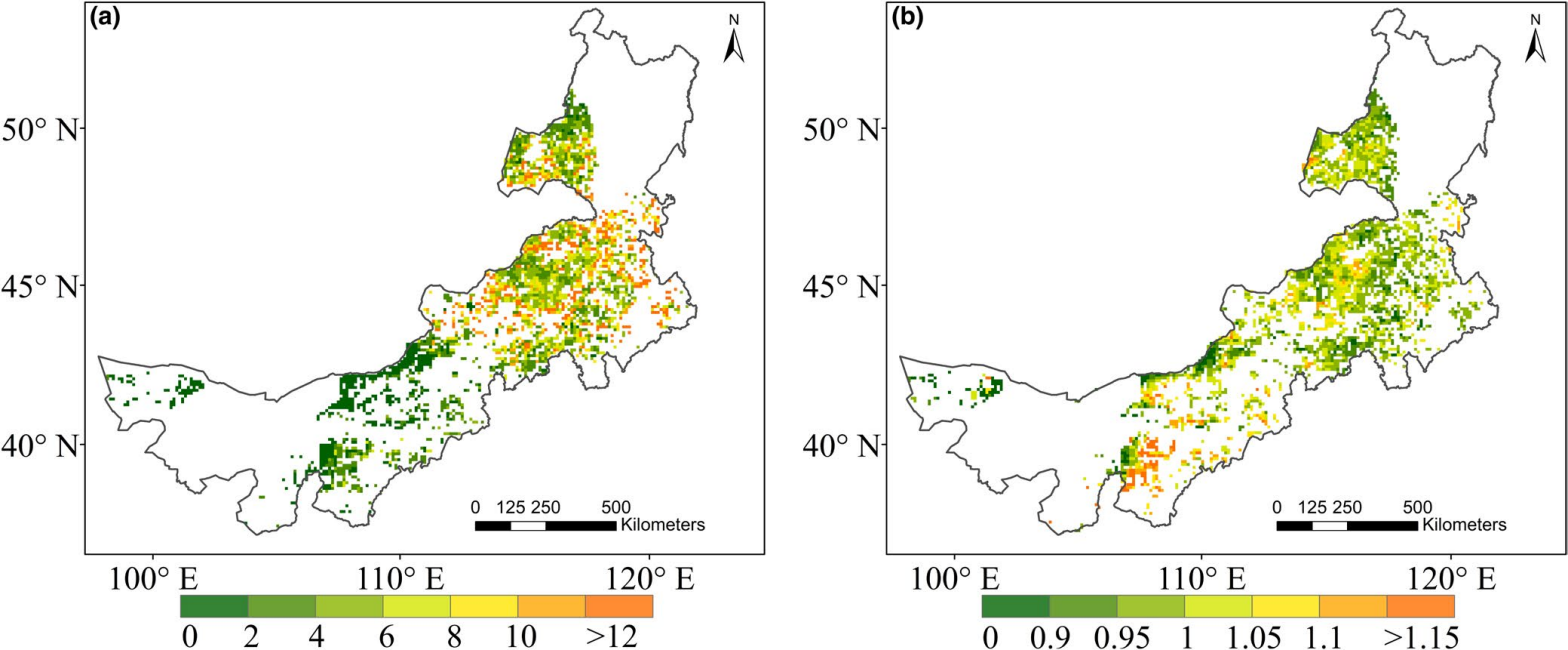
Spatial distribution of ecosystem resistance (a) and grassland resilience (b) in response to the decade‐long dry period

Apparently, ecosystem resistance to the dry period exhibited low values in the drier west area but high values in the wetter east area, suggesting that ecosystems in the drier regions was less resistant to the dry condition (Figure 5a). Especially in western regions, despite that the grasslands experienced slighter precipitation reduction, they behaved a more vulnerable response. In terms of resilience, except for the most western arid areas showing relatively low resilience, the spatial pattern of resilience was heterogeneous, without a notable spatial pattern in most areas (Figure 5b).

**FIGURE 6:**
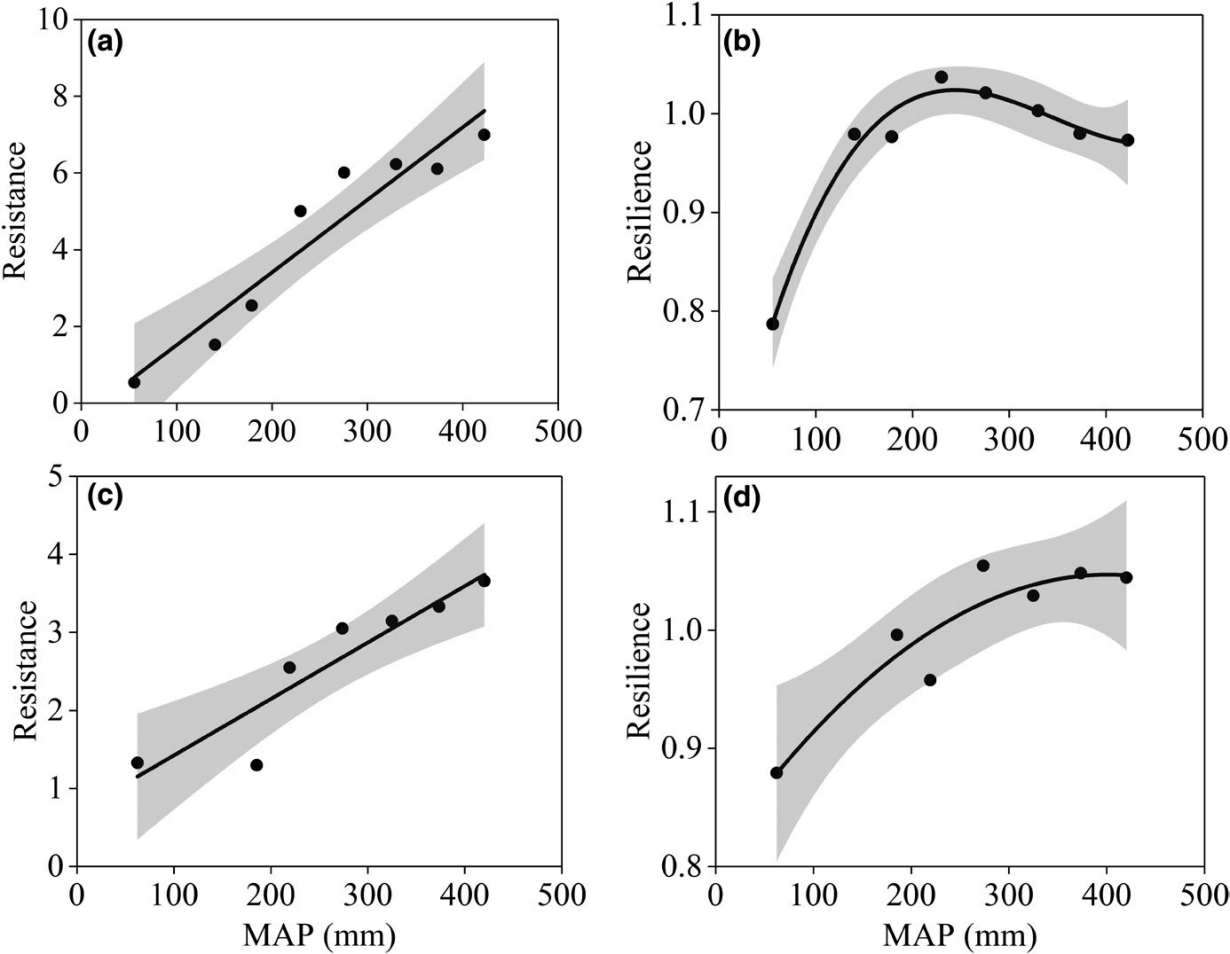
50 mm‐bin averaged resistance (a, c) and resilience (b, d) along mean annual precipitation gradient. All sites experienced rainfall reduction were used in (a) and (b), and only the sites experienced a similar degree of reduction (5%–10%) were used in (c) and (d). The 100–150 mm bin was missing in (c) and (d), because there were no sites that experienced the reduction (5%–10%). The solid lines are the best fitted lines (*p* < 0.05). The shaded area represents the 95% confidence space

Ecosystem resistance to the dry period showed an increasing trend along with the precipitation gradient (Figure 6a; *p* < 0.05; *R* ^2^ = 0.89), suggesting that grasslands in wetter climate had stronger capability to buffer the long‐term dry condition than that in drier climate. Comparing with resistance, ecosystem resilience strongly increased with MAP and peaked at MAP of 250 mm (Figure 6b; *p* < 0.05). It slightly decreased when MAP exceeded 250 mm. This indicated that the vegetation recovery rate increased with MAP only when MAP < 250 mm. The above results also suggested no trade‐off between resistance and resilience when MAP < 250 mm, and a weak trade‐off when MAP > 250 mm. Similarly, with only the sites experienced a similar degree of rainfall reduction (e.g., rain reduction between 5% and 10%), resistance increased with MAP (*p* < 0.05; *R* ^2^ = 0.85) and resilience increased first and then tended to leveled off at the wet end (Figure 6c,d; *p* < 0.05; *R* ^2^ = 0.75). This suggests that, despite experiencing a similar degree of dry condition, the drier sites exhibited both lower resistance and resilience.

## 4 DISCUSSION

### 4.1 Resistance of grasslands to the decade‐long dry period along precipitation gradient

We found that the resistance to the dry period increased with MAP, which is contrary to our expectation that grasslands in drier climates should be more resistant to drought conditions because the plant species can be more drought‐tolerated. However, our finding is consistent with some earlier reports based on intersite comparisons (Knapp et al., 2015; Heisler‐White et al., 2009; Cherwin & Knapp, 2012). For example, Knapp et al. (2015) investigated six grassland types ranging from desert grasslands to mesic tallgrass prairie in the Central United States, finding higher ability to withstand the regional‐scale drought for the grasslands in higher MAP. Heisler‐White et al. (2009) also drew a similar conclusion that in the Central Plains Region of North America, xeric grasslands have lower resistance to extreme rainfall reduction than the mesic grasslands.

Grasslands in drier climates showing a lower resistance may be attributed to the following three reasons. First, a large fraction of plant species in the xeric grasslands are fast‐growing and short‐lived species, which cannot maintain production during drought conditions (Matos et al., 2020). Therefore, the short‐lived species would contribute greatly to the reduction of biomass production of the whole plant community during the dry period, resulting in low resistance. Alternatively, dry grasslands dominated by shallow‐rooted shrub species may have high mortality during the decade‐long dry period (Jacobsen & Pratt, 2018), resulting in less resistance than herb species. Second, as MAP increases, the importance of precipitation would become weaker and that of other resources, for example, soil nutrient would become stronger. This would lead to a decrease in the sensitivity of vegetation growth to precipitation change (Huxman et al., 2004). In consequence, the long‐term dry climate condition would have less impacts on the plant communities having lower precipitation sensitivities (e.g., the mesic grasslands). Third, plant diversity is higher in the grasslands with higher MAP (Bai et al., 2008). The compensatory effect in the species‐rich ecosystem can make the productivity of the plant community main stable and exhibit the high resistant ability to climate anomalies (Tilman et al., 1997; Loreau and Hector, 2001; Bai et al., 2004; Cleland et al., 2013).

To exclude the sites with influences by human activities, we excluded the sites with NDVI reduction <1%. We realized that the nonreduction of NDVI at some of these sites may not be influenced by human activities, but just be a result of the high vegetation resistance. Unfortunately, we cannot separate these sites from the sites with influences of human activities in this study. These ecosystems with super high resistance warrant further investigation when high‐resolution land use data is available.

### 4.2 Resilience of grasslands to the decade‐long dry period along precipitation gradient

Vegetation resilience to the long‐term dry condition increased with MAP and peaked at 250 mm and then decreased slightly (Figure 6b). To the best of our knowledge, our study is the first reporting the spatial pattern of resilience in grasslands along a climate gradient. This result is beyond our expectation that ecosystems in drier climates should have higher resilience. We speculated that this spatial pattern may due to the changes in plant composition along the precipitation gradient and the duration of the dry condition. Our expectation of higher resilience in drier climate is based on the knowledge that the dominant species in grasslands are herbaceous, traits of these species in drier ecosystems favor the fast‐growing strategy after drought (Choat et al., 2018). However, here in our study, the plant communities in <250 mm are dominated by slow‐growing shrub species, whose proportion increases with aridity (Guo et al., 2012). The decade‐long dry climate would cause the mortality of the shrub species. Thereafter, the recovery of the shrub species was slow after the dry period, and thus, the slowest recovery rate occurred in the driest ecosystems. Our speculation is supported by the spatial pattern of resilience when MAP > 250 mm, where all species are herbaceous and vegetation resilience illustrated a slightly decreasing trend with MAP. In addition, as a previous study clarified, MAP of ca. 250 mm is the climatic threshold between desert steppe and typical steppe in the study region, the precipitation sensitivity of vegetation growth illustrates an opposite spatial pattern before and after this threshold (Hu et al., 2018). The fraction of herb species increases with MAP to 100% at the MAP threshold, which would result in an increase in the rate of recovery (Hu et al., 2018). However, after this threshold, recovery would be constrained since the competition of plant growth for water availability get stronger, and the importance of other resources, for example, nutrient also get greater.

### 4.3 Relationship between resistance and resilience

Our results illustrated a pattern of trade‐off between resistance and resilience when MAP > 250 mm. This is consistent with previous reports on grassland ecosystems in which herbaceous species are the dominant species in the plant community (Matos et al., 2020). However, our results do not support the hypothesis of a trade‐off between resistance and resilience when MAP < 250 mm. This discrepancy may be caused by two reasons. First, the mechanisms of vegetation response to short‐term drought and long‐term dry conditions might be different. Current knowledge on the trade‐off between resistance and resilience is mostly yielded based on studies of single‐year drought (Hoover et al., 2014; Matos et al., 2020; Gazol et al., 2017). As discussed above, it is possible that the response and recovery of a plant under a decade‐long dry period and a single‐year drought would be quite different. Second, previous studies addressed the relationship between resistance and resilience at quite a few sites, with similar plant compositions, for example, herbaceous species (Stuart‐Haëntjens et al., 2018; Craven et al., 2018; Matos et al., 2020). The findings in our study are yielded with the satellite data along a >3,000 km climate gradient. The dominant plant species shift from herbaceous grass in the wettest end to the woody shrub in the driest end.

Our findings revealed that the xeric grasslands in northern China are both less resistant and resilient in response to the periodic dry condition, implying that these ecosystems are more vulnerable than the grasslands in a wetter climate. Given projected increases in magnitude and duration of drought in the future, our study may help to identify the highly vulnerable ecosystems which are more likely to lose ecosystem functions. Thus, these susceptible regions require more attention on management and conservation for the adaptation to climate change.

## 5 CONCLUSIONS

We investigated the spatial patterns of vegetation resistance and resilience in response to a decade of dry period in the temperate grasslands in China. Our results showed that the resistance to the dry period increased with MAP, resilience increased at first until at MAP of 250 mm, and then decreased slightly. We found no trade‐off between resistance and resilience in the xeric grasslands (MAP < 250 mm), thus lower resistance and resilience happen together in drier regions. This allows us to draw a conclusion that xeric ecosystems are most vulnerable to drought events, especially a long‐term dry period as illustrated in this study. Given expected increases in drought severity and duration in the coming decades, our findings may be helpful to identify the most vulnerable ecosystems in the world for the purpose of climatic adaptation.

## CONFLICT OF INTEREST

All authors state that there is no conflict of interest.

## AUTHOR CONTRIBUTIONS

**Minqi Liang:** Data curation (lead); Formal analysis (lead); Methodology (lead); Writing–original draft (lead); Writing–review and editing (equal). **Ruochen Cao:** Writing–review and editing (equal). **Kai Di:** Data curation (supporting); Formal analysis (supporting). **Daorui Han:** Writing–review and editing (supporting). **Zhongmin Hu:** Funding acquisition (lead); Project administration (lead); Writing–review and editing (lead).

## Data Availability

The data that support the findings of this study made available at https://doi.org/10.5061/dryad.gb5mkkwq3.
